# Evaluation of cardiac masses by CMR: a tertiary center experience of cases presenting with suspicion of mass by echo or CT

**DOI:** 10.1186/1532-429X-16-S1-P105

**Published:** 2014-01-16

**Authors:** Roja Tumma, Scott Feitell, Harold Litt, Yuchi Han

**Affiliations:** 1 Radiology and imaging, Hospital of University of Pennsylvania, Philadelphia, Pennsylvania, USA; 2 Cardiology, Drexel University College of Medicine, Philadelphia, Pennsylvania, USA; 3 Cardiology, Hospital of University of Pennsylvania, Philadelphia, Pennsylvania, USA

## Background

To evaluate the effectiveness of Cardiac Magnetic Resonance Imaging (CMR) in evaluation of cardiac masses. Cardiac masses are a rare but important finding on cardiac imaging. Differentiating benign from malignant masses is crucial as an accurate diagnosis can affect the work up and management of the patient. To date very few studies have evaluated the effectiveness of CMR in aiding the diagnosis of mass found by echo or CT.

## Methods

A retrospective review of CMR performed from January 2000 to June 2013 was conducted with waiver of consent which was approved by our institutional review board. Analysis of a total of 249 consecutive patients who underwent CMR for evaluation of mass found on echo or CT were included. Follow up care, including any pathology or future imaging, was then correlated to these findings. All patients underwent CMR on a 1.5 Tesla scanner with EKG gating following the same protocol.

## Results

**Figure 1:**
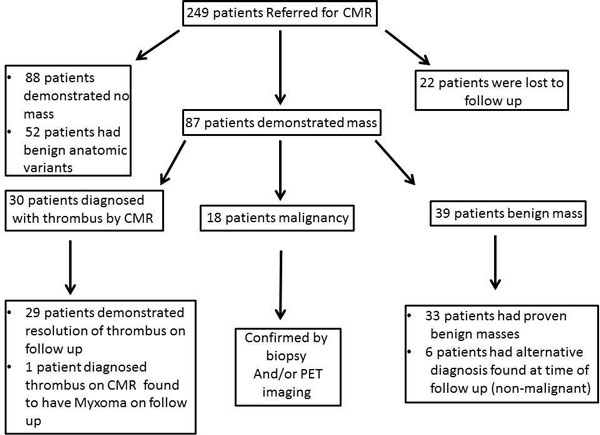


**Figure 2:**
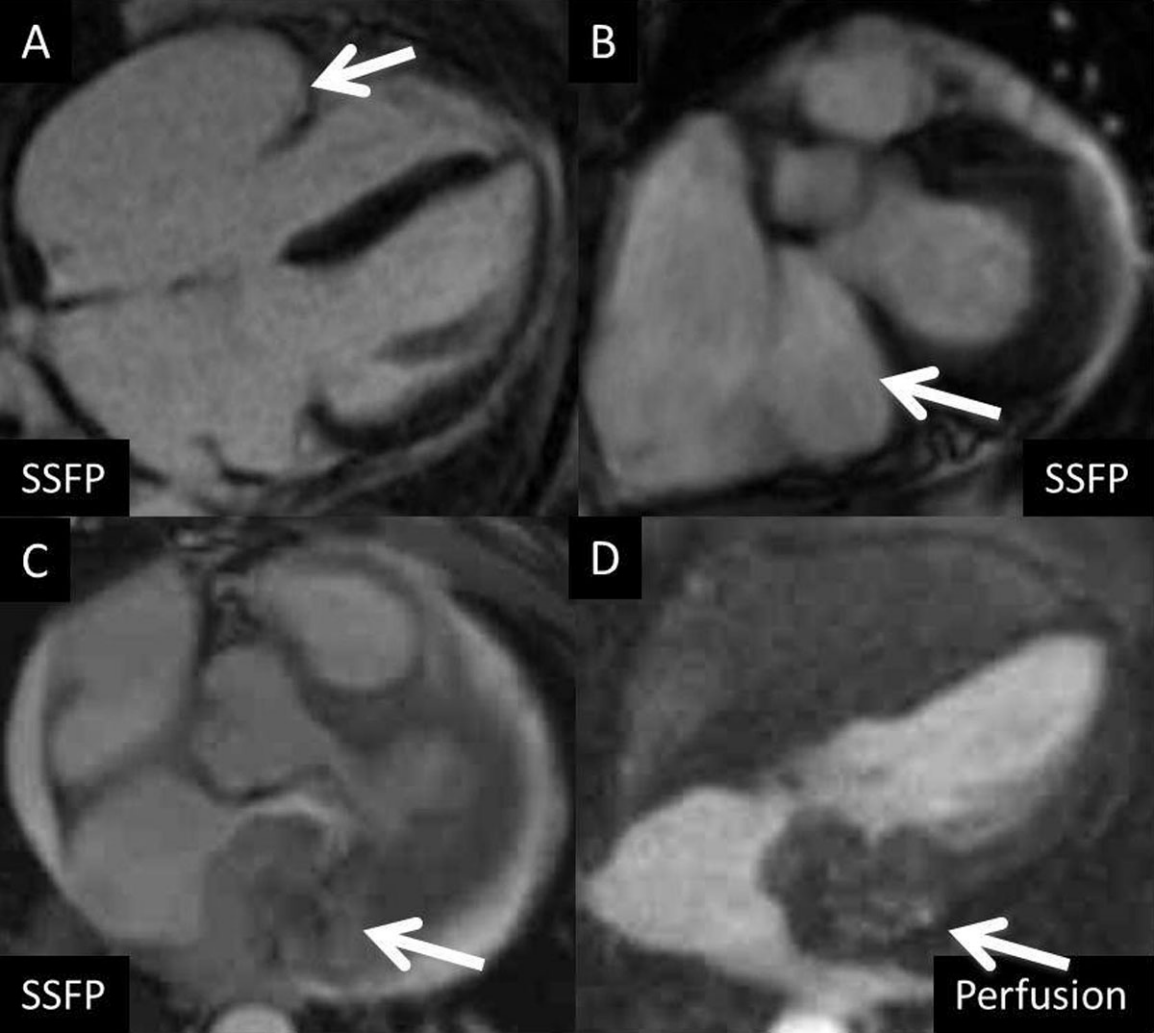
**Images A-B: Patient with large right atrial appendage simulating mass on echo; Images C-D: Patient with carcinoid tumor involving the left atrium and A-V Groove Demonstrating heterogenous early enhancement on perfusion**.

Of 249 patients referred for CMR, 22 patients with evidence of mass on CMR were lost to follow up and could not be included in the analysis. Of the remaining 227 patients, 88 patients (39%) were found to have no evidence of mass. Fifty-two patients (23%) were found to have benign anatomic variants. Of the 87 patients (38%) with evidence of mass on CMR 18 were diagnosed accurately as malignancy (12 primary cardiac tumors, 6 metastatic tumors), and were confirmed by biopsy and/or PET scan, while the remaining 69 patients had features of either a benign mass or thrombus and were managed accordingly (Figure 1). Of the 133 patients with no mass or anatomical variants, 65 had further follow up, which revealed 1 benign mass that was not seen on MRI. Sixty-eight patients did not have follow up at our institution. A case of an anatomic variant (giant right atrial appendage) and a case of malignant carcinoid are shown in Figure 2

## Conclusions

Cardiac tumors, while rare, remain an important and technically difficult diagnosis to make by current imaging modalities. We demonstrate that CMR can play a key role in making an accurate diagnosis. In our consecutive case series, over 62% of patients referred for mass evaluation were found to have either no evidence of mass or benign anatomical variants, thus saving patients further invasive work up and potential radiation exposure. 75 patients with no evidence of mass or benign variants did not have further follow up at our institution. 65 patients who did have follow up, only one patient was noted to have a benign mass persistent on subsequent imaging, but missed on CMR due to artifact. CMR also provided 100% sensitivity in the evaluation of malignant masses, making it a very effective screening tool in an oncologic work up when indicated. CMR's ability to rule out mass and/or to identify benign anatomical variants which simulate mass on echo can be invaluable. Additionally, in the presence of a mass, CMR can provide accurate differentiation of benign and malignant lesions

## Funding

None.

